# Low-Cost 3D-Printed Temporal Bone Models for Surgical Simulation: A Pilot Study

**DOI:** 10.31486/toj.26.0035

**Published:** 2026

**Authors:** Kaitlyn Tholen, Colin Curtis, Korak Sarkar, Gauri Mankekar

**Affiliations:** ^1^ School of Medicine, Louisiana State University Health Sciences Center at Shreveport, Shreveport, LA; ^2^ Ochsner BioDesign Lab, Ochsner Clinic Foundation, New Orleans, LA; ^3^ Department of Otolaryngology/Head and Neck Surgery, Louisiana State University Health Sciences Center at Shreveport, Shreveport, LA

**Keywords:** *Mastoidectomy*, *neurotology*, *printing*, *printing–three-dimensional*, *simulation training*, *temporal bone*

## Abstract

**Background:**

Surgical simulation requires realistic models for proper surgical preparation. To address the scarcity, storage complexity, and cost of cadaver specimens, alternative approaches such as three-dimensional (3D)-printed models have been developed, but 3D-printed models can be expensive and require specialized equipment and expertise. We developed and tested 3D-printed temporal bone models made with accessible, affordable materials and evaluated their use as training replacements.

**Methods:**

Using computed tomography scans of 1 adult patient and 1 pediatric patient, we 3D-printed 4 adult and 4 pediatric temporal bone models with the following materials: acrylonitrile butadiene styrene, polylactic acid, high-impact polystyrene, and Formlabs white resin (Formlabs Inc). Eight otolaryngology residents with prior experience with temporal bone drilling participated in the study. Residents were randomized to either an adult or pediatric bone and blinded to the 3D-print material used. Immediately after drilling the 3D models, the participants completed an 8-item Likert scale questionnaire that evaluated the value, ease of use, safety, and similarity of the 3D-printed temporal bones compared to cadaver temporal bones.

**Results:**

Residents rated the models as similar to cadaver bones in terms of training value but rated the bony anatomy of the models as suboptimal, specifically the mastoid air cells and dust from drilling. Statistical analysis found no significant differences across materials or residency levels (*P*>0.05). The total cost of the materials was $33.88 (US dollars), with pediatric models ranging from $2.58 to $3.03 and adult models ranging from $4.42 to $8.19.

**Conclusion:**

With improvements to the bony anatomy of our models and further optimization of the low-cost materials to improve haptic realism, 3D-printed temporal bones may provide comparable training alternatives to cadaver bones that are less costly per model and require no costly storage. Future studies should evaluate 3D-printed temporal bone models in larger within-subject cohorts and assess cost-effectiveness to determine their value relative to cadaver specimens.

## INTRODUCTION

Surgical simulation is a critical component of medical education, and realistic models are required for proper surgical preparation. The objective of surgical simulation is for trainees to be able to transfer skills learned from high-quality training into the operating room, as well as to prepare residents and physicians to be more efficient and precise in complex or rare anatomic cases.^1-3^

Globally, access to adequate surgical training and simulation-based tools is uneven, particularly in low- and middle-income countries. A 2024 review found that because access to cadaver specimens can be limited in these countries, simulation training can be beneficial.^4^ However, cost and implementation barriers often restrict the technology and models available.^5^ These limitations demonstrate the need for affordable, reproducible models that could be used across diverse training environments to help standardize training and education worldwide.

In the field of otolaryngology, the temporal bone is a particularly complex structure that requires robust, realistic surgical training for procedures such as mastoidectomy. A mastoidectomy generally requires removal of a segment of the mastoid cortex and air cells to access the middle or inner ear or to address disease. Physicians must have in-depth knowledge of the anatomy of the temporal bone and develop dexterity with the surgical drill to effectively perform such procedures and avoid damaging critical structures. Inadequate training can increase the risk of complications, including injury to the sigmoid sinus and facial nerve damage.^6^

Currently, cadaver temporal bones remain the gold standard for otolaryngology surgical simulation, and otolaryngology residents have correlated training with cadaver specimens to improved surgical performance.^6-8^ Cadaver specimens, however, are often expensive (in US dollars, up to $500 for a pair) and in short supply.^9,10^ Additionally, cadaver specimens can be associated with ethical ambiguities concerning consent and handling of human tissue, and inconsistencies in preservation and storage can greatly impact specimen reliability in surgical training.^11,12^ Further, exposure to formaldehyde, a common preservative used in cadaver specimens, poses potential health risks for trainees, including acute irritation and chronic toxic effects.^12,13^

The high cost and limited availability of cadaver specimens have led to alternative approaches in otolaryngology education and surgical simulation. Three-dimensional (3D) printing of models has been explored as an adjunct or replacement for cadaver specimens, and studies have demonstrated the potential utility of 3D-printed temporal bones for surgical simulation and otolaryngology education, including complex and patient-specific procedures.^14,15^ While 3D-printed models provide high-fidelity alternatives to cadaver specimens, 3D printing is associated with safety concerns. Thermoplastics such as acrylonitrile butadiene styrene (ABS) plastic filament and polylactic acid (PLA) filament can release ultrafine particles and volatile organic compounds when printed, and long-term exposure to these particles may cause respiratory issues.^16-18^ Wearing proper protective equipment is recommended to minimize exposure during the printing process.^18^ While most research has focused on emissions generated during the 3D printing process,^19^ some studies suggest that the finished models may continue to release volatile organic compounds or ultrafine particles under certain conditions,^16^ but the potential for exposure during postproduction handling or drilling of 3D-printed models has not been well characterized.

As 3D printing becomes more widely used in otolaryngology, efforts have been made to standardize the methods used for model development. The American Academy of Otolaryngology–Head and Neck Surgery conducted a multi-institutional analysis (published in 2021) and determined that models printed with standard resin and a stereolithography (STL) printer scored the highest in anatomic accuracy and cost approximately $10 per model.^20^ The cost of materials, however, can increase dramatically with efforts to match the soft tissue anatomy and the drilling similarity of temporal bone cadaver models. Rose et al blended multiple 3D-printing materials to accurately mimic soft tissue and bony anatomic structures, and although residents highly rated the models when they were drilled, the price per model was approximately $400.^21^ The tradeoff between model fidelity and affordability creates a challenge for institutions to choose either highly expensive printers that create biomimetic 3D-printed models or affordable printers that create single-material models.

Finding a 3D-printing material that is both affordable and anatomically accurate is crucial to developing a model that could supplement cadaver bones in surgical training. Several studies have investigated low-cost 3D-printing materials (approximately $2 or less per model) for temporal bone simulation. Gadaleta et al demonstrated that temporal bones printed with PLA were rated similarly to cadaver specimens in several domains of surgical simulation.^22^ Mowry et al found that ABS models reasonably mimicked mastoid drilling but lacked realistic mastoid air cells and drill acoustics.^23^ Kavanagh et al compared PLA, ABS, and high-impact polystyrene (HIPS) in laryngeal models and reported that HIPS provided greater flexibility and softness in surgical simulation than the PLA and ABS models, but the softness of the HIPS model was not appropriate for simulating soft tissue–like properties.^24^

To determine which low-cost material might be a sufficient substitute for cadaver bone and produce the most realistic and cost-effective temporal bone training model, we created 3D-printed temporal bones from ABS, PLA, HIPS, and Formlabs white resin (Formlabs Inc) and recruited otolaryngology residents to evaluate the utility of the models for training.

## METHODS

In compliance with ethical standards, this project was approved by the Louisiana State University Health Sciences Center at Shreveport Institutional Review Board (STUDY00002071). A waiver of consent was approved as no identifiable personal or private information was accessed and recorded for our participants.

**Figure 1.**
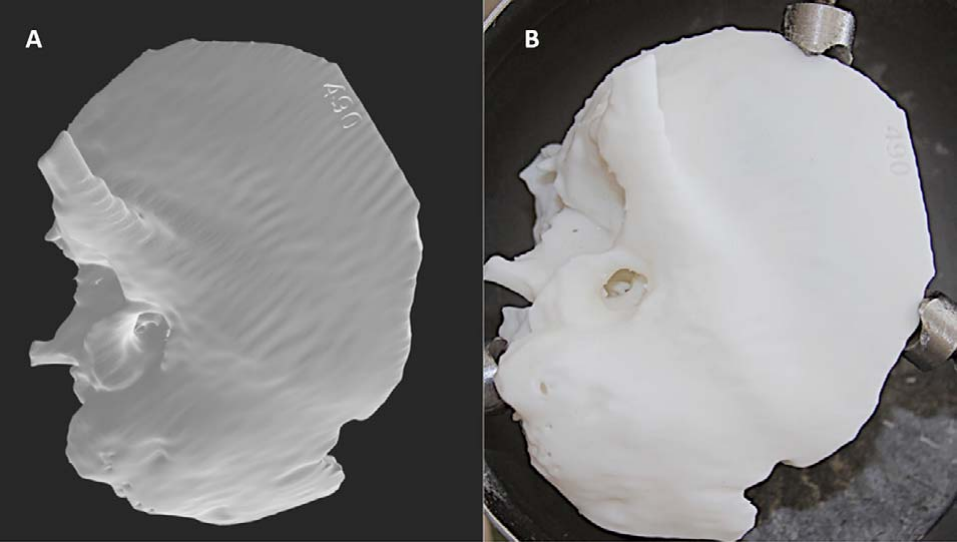
(A) Three-dimensional (3D) stereolithography object for an adult temporal bone compared to (B) the final 3D-printed model. The model was printed with Formlabs white resin (Formlabs Inc).

In collaboration with the Ochsner BioDesign Lab in New Orleans, Louisiana, 3D-printed temporal bone models were created using low-cost materials previously evaluated for their affordability and realism in surgical simulation. Imaging was obtained and deidentified for 1 adult patient and 1 pediatric patient in conjunction with the Ochsner Health Department of Radiology. The Materialise Mimics Innovation Suite (Materialise NV) software processed Digital Imaging and Communications in Medicine (DICOM) data and created a 3D STL object. The STL object was then rendered on an UltiMaker S5 Fusion Deposition Modeling (FDM) printer (UltiMaker B.V.). We created 4 adult and 4 pediatric temporal bones using affordable materials analyzed in previous studies: ABS, PLA, HIPS, and Formlabs white resin.^20,22-24^ Figure 1 shows the STL object for an adult temporal bone and the final 3D-printed Formlabs white resin model. In US dollars, the raw materials cost for the pediatric temporal bones was $2.58 for the ABS model, $2.65 for the HIPS model, and $3.03 each for the PLA and Formlabs white resin models. The raw materials cost for the adult temporal bones was $4.42 for the ABS model, $4.59 for the HIPS model, $5.39 for the PLA model, and $8.19 for the Formlabs white resin model. The total cost of the raw materials for all 8 models was $33.88. These costs represent raw printing materials only and do not include printer purchase or maintenance, postprocessing equipment, electricity, or personnel time.

Eight otolaryngology residents with previous temporal bone drilling experience were recruited to evaluate the models: 3 second-year residents (postgraduate year [PGY] 2), 2 third-year residents (PGY3), 1 fourth-year resident (PGY4), and 2 fifth-year residents (PGY5). Participants wore appropriate personal protective equipment, including gloves and surgical masks. The temporal bone laboratory at Louisiana State University Health Sciences Center at Shreveport is equipped with general building ventilation, and localized suction was provided at the drilling site to remove model dust and debris. Each resident was provided with instructions to perform a simple mastoidectomy on the 3D-printed temporal bone model. Residents were randomized to either an adult or pediatric bone and blinded to the 3D-print material used.

**Figure 2.**
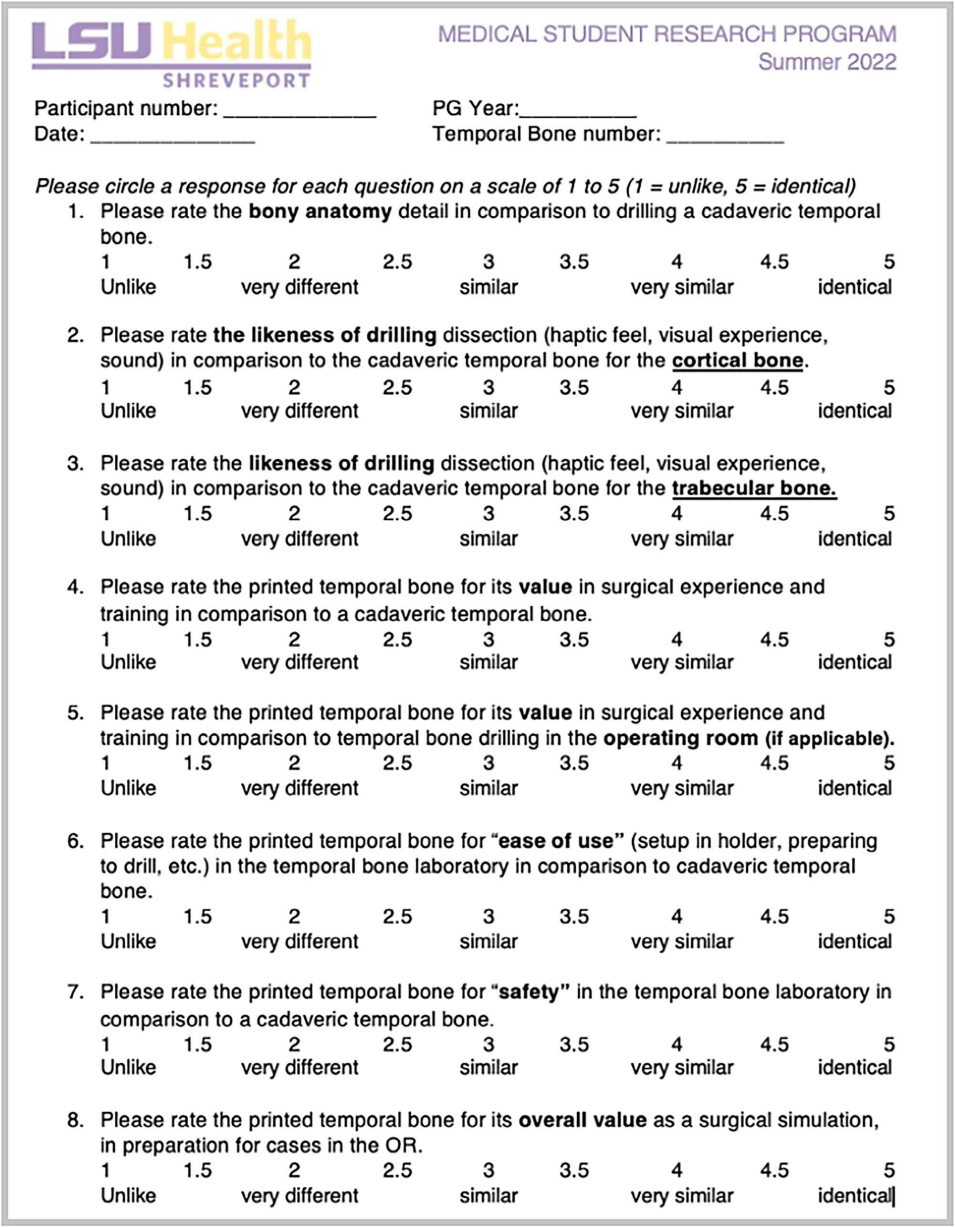
The survey comparing the 3D-printed models to cadaver bones was adapted from Gadaleta et al^22^ and given to otolaryngology residents after they drilled the 3D-printed temporal bone models. Responses were on a scale of 1 to 5, with 1 corresponding to unlike cadaver bone and 5 corresponding to identical to cadaver bone.

Immediately after drilling the 3D-printed models, participants completed an 8-item Likert scale questionnaire (Figure 2) adapted from Gadaleta et al.^22^ Our intent was to replicate the Gadaleta et al study to allow direct comparison of results, so the Gadaleta et al study served as the survey pilot for our study. The questionnaire evaluated the value, ease of use, safety, and similarity of the 3D-printed temporal bones compared to cadaver temporal bones, with a score of 1 meaning the 3D-printed model was unlike a cadaver bone and a score of 5 meaning the model was identical to a cadaver bone. While these categories are broad in scope, we intentionally matched the domains assessed in the Gadaleta et al study.^22^

### Statistical Analysis

Survey results are summarized using means and standard deviations to maintain consistency with the Gadaleta et al study.^22^ We chose to analyze our data as ordinal variables rather than continuous variables because the distance between Likert scale responses may not be equal. Nonparametric Kruskal-Wallis and Mann-Whitney *U* tests were used to compare responses across groups, and effect sizes (omega squared and rank-biserial correlation) were calculated to assess the magnitude of differences. The omega squared analog (ω^2^) estimates the proportion of variability in survey responses attributed to the grouping variable, with values of approximately 0.01, 0.06, and 0.14 corresponding to small, medium, and large effects, respectively. For rank-biserial correlations (r), values closer to 0 indicate minimal differences between groups, whereas values approaching +1 or –1 indicate larger group differences. Statistical analyses were conducted with the assistance of an artificial intelligence–based data analysis tool.

A power analysis determined that for a within-subject cohort study (4 materials per resident), 125 residents would be needed to detect significant differences (Cohen f=0.25, 80% power). Because of limited resident availability and because our study was an unfunded, single institution pilot, this study was underpowered for detecting statistical differences in the survey. As such, all *P* values and effect sizes are interpreted as exploratory.

## RESULTS

**Figure 3.**
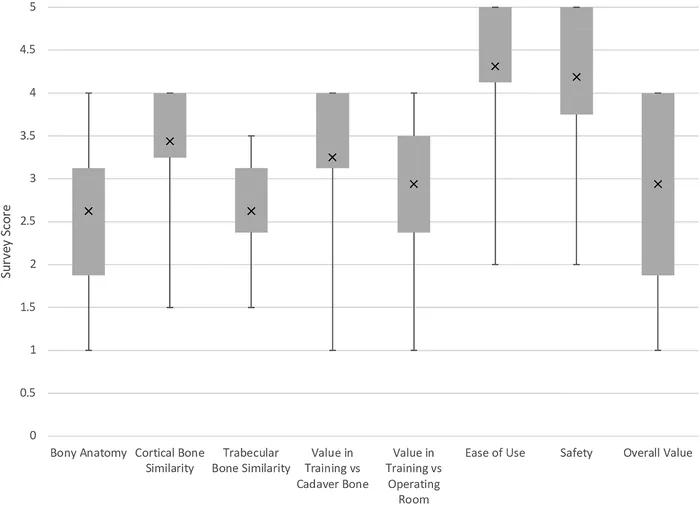
Results of the Likert-scale questionnaire are expressed in a box plot. The × represents the average score for each question.

The results of the Likert scale survey are presented in Figure 3. The average rating for overall value of drilling the 3D-printed bones was 2.94 ± 1.24. The 3D-printed models were highly rated for their ease of use (4.31 ± 1.16) and their perceived safety (4.19 ± 1.13) in the laboratory compared to cadaver bones. Residents rated the bones “similar” to cadaver bones in terms of their likeness to drilling cortical bone (3.44 ± 0.94), but they were rated “very different” in their likeness to drilling trabecular bone (2.63 ± 0.69). The 3D-printed bones were rated “similar” to cadaver bones for their value in training (3.25 ± 1.13) but were rated lower than operating room training (2.93 ± 1.02). The 3D-printed bones were rated poorly for their bony anatomy compared to cadaver bones (2.63 ± 1.03).

All *P* values and effect sizes are reported in Tables 1, 2, and 3. We conducted a Kruskal-Wallis test to assess differences in survey responses across the 4 bone materials and across PGY levels (Table 1). No statistically significant differences were found for any survey question (*P*>0.05 for all comparisons). The effect size estimates reported in Table 1 should be interpreted as exploratory because of the small sample size.

**Table 1.** Kruskal-Wallis *P* Values and Omega Squared Effect Sizes for Survey Responses Across Bone Materials and Postgraduate Year Levels

	3D-Printed Bone Material		Postgraduate Year Level	
Survey Question	Kruskal-Wallis *P* Value	Omega Squared Effect Size	Kruskal-Wallis *P* Value	Omega Squared Effect Size
Bony anatomy	0.38	0.02	0.59	0.02
Cortical bone similarity	0.09	0.04	0.06	0.43
Trabecular bone similarity	0.55	0.01	0.69	0.01
Value in training vs cadaver bone	0.32	0.02	0.51	0.01
Value in training vs operating room	0.44	0.01	0.45	0.01
Ease of use	0.72	0	0.77	0
Safety	0.81	0	0.83	0
Overall value	0.65	0.01	0.48	0.02

**Table 2.** Mann-Whitney *U* Test *P* Values and Rank-Biserial Correlations (r) Comparing Each 3D-Printed Bone Material to the Other 3D-Printed Bone Materials

	ABS vs Others		PLA vs Others		HIPS vs Others		Resin vs Others	
Survey Question	*P* Value	r	*P* Value	r	*P* Value	r	*P* Value	r
Bony anatomy	0.77	–0.01	0.41	–0.05	0.85	–0.01	0.73	–0.01
Cortical bone similarity	0.88	–0.01	0.67	–0.03	0.88	–0.01	0.91	0
Trabecular bone similarity	0.54	–0.03	0.39	–0.05	0.71	–0.02	0.59	–0.03
Value in training vs cadaver bone	0.08	–0.1	0.59	–0.04	0.81	–0.01	0.64	–0.02
Value in training vs operating room	0.61	–0.02	0.46	–0.05	0.79	–0.01	0.76	–0.01
Ease of use	0.65	–0.02	0.44	–0.06	0.91	0	0.72	–0.01
Safety	0.74	–0.01	0.33	–0.07	0.77	–0.02	0.84	–0.01
Overall value	0.08	–0.1	0.56	–0.04	0.84	–0.01	0.66	–0.02

ABS, acrylonitrile butadiene styrene; HIPS, high-impact polystyrene; PLA, polylactic acid; resin, Formlabs white resin (Formlabs Inc).

**Table 3.** Mann-Whitney *U* Test *P* Values and Rank-Biserial Correlations (r) Comparing Each Postgraduate Year (PGY) Level to the Other Postgraduate Year Levels

	PGY2 vs Others		PGY3 vs Others		PGY4 vs Others		PGY5 vs Others	
Survey Question	*P* Value	r	*P* Value	r	*P* Value	r	*P* Value	r
Bony anatomy	0.44	–0.07	0.88	–0.01	0.82	–0.01	0.75	–0.02
Cortical bone similarity	0.09	–0.31	0.79	–0.02	0.91	0	0.69	–0.03
Trabecular bone similarity	0.49	–0.06	0.77	–0.02	0.83	–0.01	0.66	–0.03
Value in training vs cadaver bone	0.08	–0.37	0.82	–0.01	0.89	–0.01	0.74	–0.02
Value in training vs operating room	0.59	–0.05	0.85	–0.01	0.79	–0.01	0.72	–0.02
Ease of use	0.55	–0.05	0.73	–0.03	0.85	–0.01	0.68	–0.04
Safety	0.66	–0.04	0.69	–0.03	0.88	–0.01	0.71	–0.03
Overall value	0.08	–0.37	0.81	–0.02	0.92	–0.01	0.73	–0.03

Mann-Whitney *U* tests comparing each 3D-printed bone material to the other 3D-printed bone materials did not reveal any significant findings (Table 2). Mann-Whitney *U* tests comparing each PGY level to the other PGY levels did not reveal any significant findings (Table 3). Rank-biserial correlation (r) effect sizes reported in Tables 2 and 3 provide descriptive estimates of the magnitude and direction of group differences.

## DISCUSSION

In previous studies, 3D-printed models have been reported to be a successful adjunct to training with cadaver bones.^2,3,14,15^ Our study suggests that 3D-printed temporal bones may serve as easy-to-use and practical training tools for residents in surgical preparation. Overall, residents rated the models as similar to cadaver specimens in terms of ease of use, perceived safety during drilling, likeness to cortical bone, and value in training; however, ratings for trabecular bone realism and overall bony anatomy were lower, highlighting persistent challenges in re-creating accurate haptic feedback using low-cost printing materials. Material-specific differences were also observed, with residents rating models constructed of PLA lowest across several domains, while models constructed of Formlabs white resin produced drilling dust that was most similar to true bone dust. Junior residents (PGY2) reported greater educational value than senior (PGY3 through PGY5) residents, suggesting that these models may be particularly useful during earlier stages of surgical training. Although no statistically significant differences were identified between materials or PGY levels, this pilot study was underpowered for definitive subgroup comparisons.

The low ratings for trabecular bone similarity may be because the 3D-printing material is homogenous and might not accurately replicate the transition to and difference from cortical bone. The low ratings for bony anatomy could be because mastoid bone air cells in our pediatric and adult models were absent or poorly formed. This finding aligns with a previous report of problems with creating accurate mastoid air cells^25^ and limits the ability of a 3D-printed model to provide the same haptic feedback as a cadaver bone. Advancements in 3D printing, such as multimaterial printing techniques, could improve the accuracy of varying bone textures and haptic feedback during drilling^24^; however, multimaterial printing requires more expensive printers and increases the overall cost of the models,^21^ which may limit feasibility for widespread adoption in training programs.

In our study, the overall value of the 3D-printed bones was rated as 2.94 ± 1.24, which is lower than the overall value rating in the Gadaleta et al study (4.55 ± 0.72).^22^ However, the 3D-printed bones in the Gadaleta et al study incorporated color-coded neural structures, such as the facial nerve and chorda tympani, to provide anatomic orientation and enhanced visual feedback during drilling.^22^ Our models only included the bony structures of the middle ear without colored neural structures, which may have made it harder for residents to visualize critical anatomy, especially for participants with less drilling experience. Some studies have investigated optimizing 3D printing for different anatomic structures, but printer and material costs are extremely high to mimic bone, soft tissue, and nerves.^20,21^ Another key difference between our study and the Gadaleta et al study^22^ is our use of 4 low-cost materials (ABS, PLA, HIPS, and Formlabs white resin) vs PLA only. Our average rating for overall value therefore reflects all materials and may help explain the difference in findings.

**Figure 4.**
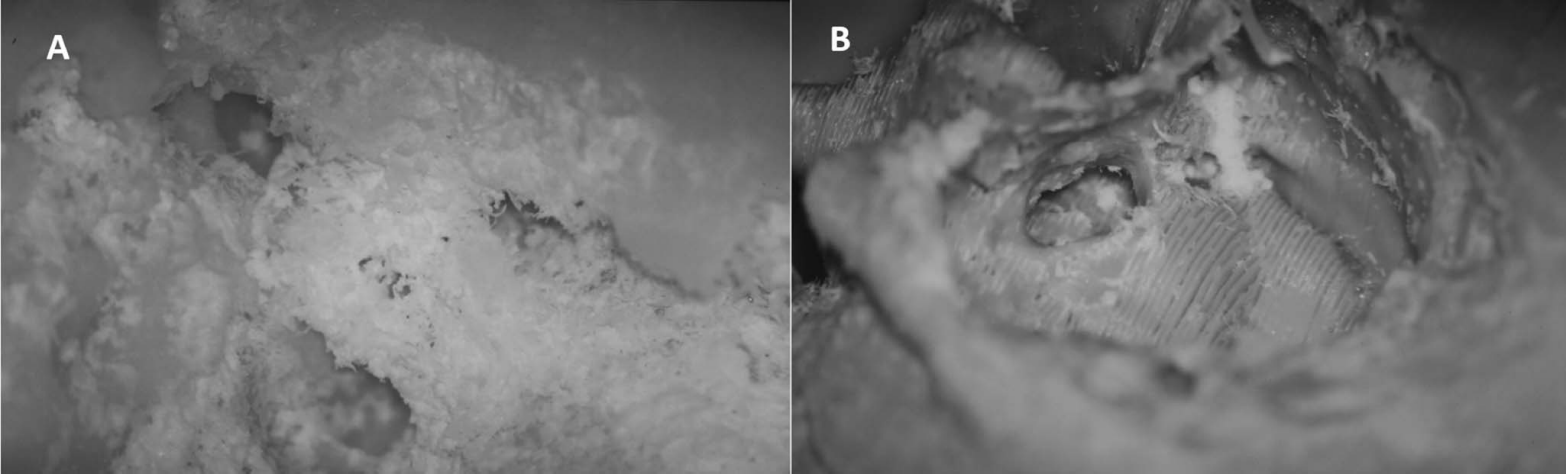
Postdrilling microscopic images demonstrate the difference in bone model dust and material texture of (A) Formlabs white resin (Formlabs Inc) and (B) acrylonitrile butadiene styrene.

Previous studies demonstrated that ABS and PLA were cost-effective materials (approximately $2 per model) that adequately mimicked temporal bone drilling.^22,23^ In our study, residents rated PLA as the worst material in terms of ease of use, safety, similarity to trabecular bone, and value in training compared to cadaver specimens and to training in the operating room. These findings contrast with the findings of Gadaleta et al^22^ whose participants rated PLA very highly. Our residents also noted that ABS created dust that clumped and flaked off during drilling unlike true bone dust (Figure 4), impacting the tactile feedback. Residents reported similar drilling characteristics with PLA.

Further research is needed to understand the health implications of exposure to this dust in training environments, considering that these materials release ultrafine particles and volatile organic compounds when being printed.^16-18^ Although standard personal protective equipment and suction were used during drilling, we did not assess how well our laboratory filtered ultrafine particles and volatile organic compounds during drilling. Future studies should examine the potential health risks associated with drilling 3D-printed models vs cadaver specimens and implement proper filtration systems to mitigate any risks.

Residents noted that drilling the 3D-printed models made of Formlabs white resin created dust that was most like bone (Figure 4), but Formlabs white resin is slightly more expensive than ABS and PLA (approximately $10 vs $2 per model).^20^ In our study, the adult temporal bone printed with Formlabs white resin was more expensive than the other adult models ($8.19 vs $4.42, $4.59, and $5.39). Despite this higher cost, providing all the otolaryngology residents in our program with 1 adult temporal bone printed with Formlabs white resin (total cost of $122.85) would be cheaper than providing each resident with a cadaver specimen (up to $500 per pair for a total cost of $4,000).^9,10^ While our findings suggest that Formlabs white resin offers more anatomic realism and affordability than the other materials we tested, more research is needed to determine the best use of cost-effective materials to optimize surgical training. Importantly, while the individual models are inexpensive to make, institutions without a 3D-printing program will have initial costs of purchasing a printer and the necessary materials. The cost of the printer we used, an UltiMaker S5 FDM printer, ranges from $6,000 to $7,000, depending on the vendor and configuration. These upfront costs may influence an institution's ability to adopt 3D-printed surgical training models.

Three-dimensional–printed models have been shown to be useful adjunct training options for medical students and residents and may provide advantages over computed tomography images alone in surgical preparation.^2,3^ Our junior residents gave higher ratings on value in training compared to senior residents, which supports the suggestion in previous studies that 3D-printed temporal bone models are possibly better suited for junior residents or medical students to learn hand positioning and steadiness, drilling techniques, and bony anatomy.^26^ By incorporating 3D-printed temporal bones into early education, training programs could potentially reserve cadaver bones for later stages of training. A formal cost-effective analysis of 3D-printed models and an evaluation of the skills transfer from 3D-printed bones to surgical outcomes have not yet been done.^27^

While we adapted our survey from Gadaleta et al,^22^ several questions could be added to further determine the level of user satisfaction with the model. For example, McMillan et al divided bony anatomy questions into the contour and drilling of each part of the bone, thereby obtaining a more precise view of the weaknesses of the 3D-printed model.^28^ Our questionnaire could have included questions regarding the bony anatomy of specific structures vs overall, the irritation caused by the 3D model dust, and the level to which the material dust clogs the suction compared to normal bone dust. Such specific details would better clarify the value and realism of inexpensive, nonbiomimetic models compared to expensive, biomimetic 3D-printed models or cadaver bones. A further limitation is that our survey instrument, although adapted from a published study, has not undergone formal validation, and several domains are broad and may not capture the specific anatomic or haptic attributes that influence model performance. Additionally, when calculating average scores, we weighted all survey questions equally according to the methodology of the prior study, which may not account for the varying importance of each factor in surgical training. Future studies could improve upon this approach by piloting a detailed and validated survey and incorporating weighting schemes determined by consensus from attending surgeons. Although we recruited residents with varying drilling experience (ie, PGY2 residents who had drilled <5 cadaver temporal bones vs PGY5 residents who had more experience), participants should be asked to provide the number of cadaver specimens they have drilled to quantify how experience affects the perception of the 3D-printed models.

Our study has several limitations. While a power analysis determined that 125 residents would be needed to detect significant differences in a within-subject cohort study, our project was pending funding to expand, so we were limited to a small number of residents and a small number of 3D-printed bones. Ideally, residents should have drilled bones of each material to allow for within-subject comparisons and stronger conclusions about the materials. Additionally, we only used 1 adult and 1 pediatric scan to create our bones, limiting the anatomic diversity of our models. Future studies should include multiple scans across age, sex, and pathology to ensure model selection is generalizable. Another limitation is that our study relied solely on subjective resident survey ratings without objective measures such as accuracy or error rates that would provide a more robust assessment of 3D-printed model value. Resident survey responses could have been affected by observer bias and influenced by prior training experiences or expectations. We did not include attending physicians because of the limited number of 3D-printed bones, so future studies should incorporate the perspectives of experienced surgeons to provide a more comprehensive analysis of how useful 3D-printed bones are at all levels of surgical training.

While our models may be valuable for foundational training in temporal bone drilling, their applicability to advanced otologic procedures may be limited without the incorporation of multimaterial printing and labeled nerves and soft tissue. Further work could explore if these modifications provide value for advanced otologic surgical preparation.

This pilot study did not quantify the objective impact that the 3D-printed bones have on performance in the operating room. In a review of 3D-printed models used in orthopedic surgeries, multiple studies demonstrated improvements in operating time, blood loss, accuracy, and other measures in the operating room.^29^ A comprehensive study should assess surgical performance in the operating room or compare cadaver bone drilling before and after using 3D-printed temporal bones to determine whether these models provide similar training benefits.

## CONCLUSION

Our pilot study demonstrated that 3D-printed temporal bones may serve as practical and easy-to-use adjuncts to cadaver bones. With improvements to the bony anatomy and further optimization of low-cost materials to improve haptic realism, 3D-printed temporal bones could become promising, cost-effective tools for surgical simulation that require no costly storage. Larger, within-subject cohort studies and formal cost analyses are warranted, and future studies should investigate model optimization, anatomic realism, surgical skill development, and potential safety considerations associated with model drilling.

## Notes

*This article meets the Accreditation Council for Graduate Medical Education and the American Board of Medical Specialties Maintenance of Certification competencies for Patient Care, Medical Knowledge, and Practice-Based Learning and Improvement.*
